# No Effect of a Commercially Used Odor Repellent for Roe Deer (*Capreolus capreolus*) Protection During Meadow Harvest

**DOI:** 10.3390/ani15192932

**Published:** 2025-10-09

**Authors:** Jan Cukor, Klára Matějka Košinová, Rostislav Linda, Vlastimil Skoták, Richard Ševčík, Tereza Červená, Kateřina Brynychová, Zdeněk Vacek

**Affiliations:** 1 Forestry and Game Management Research Institute, Strnady 136, 252 02 Jíloviště, Czech Republic; 2 Faculty of Forestry and Wood Sciences, Czech University of Life Sciences Prague, Kamýcká 129, 165 00 Prague-Suchdol, Czech Republic; 3 Faculty of Forestry and Wood Technology, Mendel University in Brno, Zemědělská 1, Černá Pole, 613 00 Brno, Czech Republic

**Keywords:** roe deer fawn, agricultural intensification, biodiversity conservation, wildlife protection, human–wildlife conflicts

## Abstract

**Simple Summary:**

In the agricultural landscapes of Central Europe, hundreds of thousands of roe deer (*Capreolus capreolus*) fawns are killed annually during meadow harvests. To prevent this particularly unethical mortality of juveniles, wildlife managers employ various protection measures. One common method is to apply odor repellents in high-risk areas before harvest. Here, we evaluated the effect of the odor repellents on treated areas relative to controls without any measures. The abundance of fawns and adult roe deer was assessed before and after odor application using unmanned aerial vehicles equipped with thermal cameras. The results showed that roe deer numbers on treated fields did not differ from those on control meadows without repellents. The expected efficacy was not demonstrated either in the short term (i.e., in the days immediately following application) or over longer intervals of several weeks. The ineffectiveness of odor repellent for protecting roe deer during meadow harvest indicates shortcomings in current practice and underscores the need to adopt alternative methods, such as thermal-imaging drones.

**Abstract:**

In Central Europe, the fawning season of roe deer (*Capreolus capreolus*) directly overlaps with meadow and alfalfa harvest, typically from late May to early June. During these operations, tens or more likely hundreds of thousands of fawns are mutilated by agricultural machinery. To mitigate this unethical mortality, wildlife managers often deploy odor repellents to drive roe deer individuals from high-risk fields before mowing. Therefore, we evaluated repellent efficacy in a paired design. The abundance of roe deer was quantified by drones equipped with thermal cameras before and after repellent application and then compared with untreated control meadows. Results showed high adult abundance that did not differ significantly among treatments. The highest median was paradoxically observed on meadows “after application” (8.25 ind./10 ha), followed by “not treated” meadows (7.92 ind./10 ha), and “before application” (5.72 ind./10 ha). For fawns, differences between treated and untreated plots were likewise non-significant. Their numbers increased over time after application, consistent with the peak of parturition in the second half of May. Overall, the study confirms that the tested odor repellent, when applied according to the manufacturer’s protocol, did not reduce roe deer presence on meadows. This underscores the need to consider alternative approaches, such as the use of thermal-imaging drones combined with the subsequent translocation of detected fawns to safe locations.

## 1. Introduction

Landscapes worldwide are heavily impacted by human activity, which is especially true for agroecosystems [1,2,3,4]. Central Europe is characterized by its highly cultivated agricultural landscape, which has substantial consequences for wildlife and other environmental aspects [5,6]. This intensive agriculture, including paraquat application and machinery use, has led to a rapid loss of biodiversity in the region [7,8,9]. Over the last few decades, the intensification of mechanized farming has accelerated harvesting practices, including meadow and alfalfa field harvest. The speed of harvesting machines typically exceeds 15 km/h and their cutting bars can be 14 m wide [10]. Accelerated meadow harvest and mowing operations negatively affect the density and diversity of invertebrate [11] and other animal populations. Especially birds nesting in agricultural land belong to the most threatened groups of animals, including endangered species [12,13,14].

The roe deer (*Capreolus capreolus* L.) is one of the most vulnerable wildlife species to agricultural machinery, especially the juveniles [15,16]. Meadow harvest can be fatal for roe deer due to the life strategy and behavior of newborn fawns. Outside of nursing time, mothers leave them in a shelter, often a grassland ready for early harvesting. Regarding fawn mortality, in south-central Sweden, it was estimated that farm machinery is responsible for 31% of the deaths of newborns during meadow harvest [17]. Lower mortality was detected in Switzerland, where 19.5% of 930 reported deaths of fawns were related to mowing activities [18]. Lower mortality rates of newborn fawns were reported from Westphalia, where losses of roe deer fawns killed by mowing were 14.5%. This corresponds to the total number of 84,000 roe deer fawns killed annually in the 1970s with much less efficient technology [19]. Therefore, the total number of killed individuals has to be more than hundreds of thousands in Central Europe.

For these reasons, various methods for protecting roe deer fawns during meadow harvest have been developed in previous decades. The oldest and one of most utilized is to walk through the vegetation with pointer dogs during the morning before mowing operations, which is a form of direct scaring. The method requires the time flexibility of a large number of participants, consistency, and the cooperation of the other party, i.e., the farmer [17]. Various scarecrows, optical or acoustical, have been tested with varying degrees of success [15]. Acoustic scarecrows are probably the least used, whether they are made of cans, various piezo sirens, ultrasonic buzzers, gas-emitting scarecrows, or commercially produced acoustical devices designed for scaring wildlife. Several forms of optical scarecrows are used—rods with aluminum foil, cans, flags, or a combination of optical and acoustical devices, i.e., acoustic-light scarecrows [20,21].

Odor repellents are another group of deterrents that are often used—with contradictory results—for a wide range of purposes, e.g., for the prevention of wildlife-vehicle collisions [22,23] or for protecting forest stands and crops against damage caused by wild ungulates [24,25]. Wild ungulates perceive scent stimuli, enabling them to detect predators and adapt their behavior accordingly. Furthermore, because it is a natural defense mechanism of prey against danger/predator [26], odor repellents are perceived as an effective form of protection or as a defense against ungulates.

Apart from odor repellents, the latest modern technology—drones equipped with thermal imaging cameras are used as well [15,27]. While the effectiveness of drones has been verified and the detection rate is nearly 100% for fawns in meadows [15], the effectiveness of odor repellents is rarely the subject of study. Insufficient research was performed, especially in the area of protection and expulsion of adult roe deer and fawns from risk areas before harvest. Therefore, the main objectives of this study are to: (i) evaluate the number of adult roe deer in the meadows treated with odor repellents and in control ones; (ii) identify the effect of the sex of the individual on the efficacy of odor repellents, and finally, (iii) evaluate the duration of efficacy of odor repellents in the protection of fawns in meadows before harvest.

## 2. Material and Methods

### 2.1. Study Area

**Figure 1:**
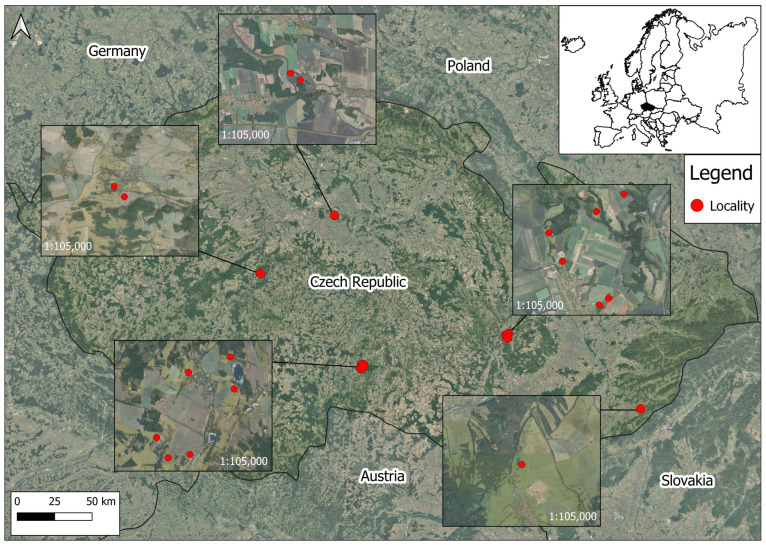
Location of experimental plots for odor repellent testing (some points overlap due to the close proximity).

Within the Czech Republic, a total of 25 research areas were designated in 2023 and 2024 to test the validity of the odor repellent. The effectiveness was tested in permanent grasslands in the Central Bohemian Region, South Bohemian Region, and Vysočina Region. The typical localities of interest were hunting districts with mixed landscape cover. In general, the Czech Republic is covered by 33.4% forest, and this is also true for the selected locations (see Figure 1). Their altitudes ranged from 180 to 600 m a.s.l., and all study areas belong to Cfb—temperate oceanic climate according to Koppen’s climatic classification. From the perspective of roe deer management, the hunting bags from the last ten years ranged from 99,828 to 124,897 [28], which corresponds to an average annual hunting bag of 18 ind./1000 ha from the hunting ground area.

### 2.2. Odor Repellent Used

The Czech prevention product set Pacholek was used to test the effectiveness of odor repellents for the protection of roe deer during hayrides. The product is composed of isovaleric acid, oleic acid, propan-2-ol, acetone, and lavender oil [29]. The product is an aerosol in a bottle with an applicator. It is delivered with a carrier made of polyurethane foam, which is formed by the polyaddition of isocyanate and polyols, showing increased resistance to photo-oxidation when applied outdoors. This foam is insoluble in water and organic solvents, and has a short-term temperature stability of up to 200 °C. Its controlled porous structure—open pores—allows for retention and gradual release of the odorant. It is biodegraded in the soil by bacteria. The shelf life of the BIO10 carrier in air is over five years [29]. The validated set contains six pieces of concentrates and 450 pieces of carriers composed mainly of starch. The package is intended for an area of 50 ha.

### 2.3. Odor Repellent Application Methodology

Out of the 25 research plots, 20 were treated according to the Prevention methodology, and further five were designated as control plots where no protection was applied. The area of each plot ranged from 1 to 12 ha (5.52 ha ± 2.47 SD in average; the specific areas of each plot are given in the results). The application procedure was carried out exactly as defined in the manufacturer’s instructions, reflecting the way this product is routinely used in wildlife management practice. The evaluated plots were monitored over two years, before and during the haying season. The areas were treated with an odor fence on average three weeks before mowing. The monitoring took place between 26 April and 5 June 2023, and between 3 May and 14 June 2024. Each carrier was injected with aerosol for approximately 1 s. The carriers were then spread on the plot at a density of 9 carriers/ha. The placement was carried out according to the manufacturer’s instructions, starting from the highest point of the area with a 40 m spacing on either side of the high point and then at a given spacing parallel to the remaining area.

### 2.4. Roe Deer Monitoring

For the unmanned aerial vehicle (UAV) thermal imaging search of roe deer, a DJI Matrice 30T UAV equipped with an integrated thermal imaging camera with a resolution of 640 × 512 px and a frame rate of 30 Hz was used. The camera can measure surface temperatures with an accuracy of ±2 °C or 2% of the measurement range, with a thermal sensitivity of ≤50 mK at 30 °C. Flights were planned using the DJI Pilot 2 app, allowing the route to be flown in its entirety without image overlap, which is essential for effective area coverage. Flight speed ranged between 4 and 6 m/s, as higher speeds make image analysis difficult and slower speeds significantly reduce terrain coverage. The integrated system allows for real-time image transmission and interfacing with GPS to accurately store the location of identified fawns.

We chose a height of 40 m above the terrain for the imaging, which represents an optimal compromise between spatial resolution and efficiency of territory coverage. The integrated thermal camera of the DJI Matrice 30T drone, with a resolution of 640 × 512 px and a diagonal field of view of 61°, covers an area of approximately 36.8 × 29.4 m from this height. Each pixel in the image corresponds to approximately 5.75 cm in real space, so an object the size of a fawn (20 × 20 cm) is shown at approximately 3.5 × 3.5 pixels. This value meets the generally recommended threshold of at least 3 × 3 pixels for reliable detection of small objects. Thanks to the integrated camera with optical zoom up to 16× (and digital zoom up to 200×), every object found could be visually inspected remotely in real time without lowering the flight altitude. This feature made it possible to verify that the animal was indeed a roe deer and to rule out confusion with different game species or similar warm objects in the field (e.g., rabbits, hares). The visual inspection with the zoom camera significantly increased the efficiency and accuracy of the entire shooting. Flights were conducted in the morning hours when the difference between the deer’s body temperature and the surrounding environment is greatest, which markedly increases contrast in the thermal image and facilitates detection.

The recording data of each individual was evaluated directly during the flight on the DJI RC Plus touchscreen controller. Positions were stored in the controller, and in addition, photos of the individuals were taken, recording the GPS position and time the image was captured for possible review and further work in GPS location-enabled software. A total of 122 flights were conducted in 2023 and 129 flights in 2024 as part of wildlife monitoring.

### 2.5. Statistical Analysis

For general overview of adult abundance data, a boxplot illustrating the relative adult abundance (ind./10 ha) for each study location is presented. Locations with repellent application and without repellent application are distinguished by a different color in the boxplot.

The effect was assessed by comparing relative adult abundances before and after repellent application, as well as recording data from a location without treatment (where repellent was not applied). For the “before” variant, all drone flight records before the repellent application date were considered. The same was performed for the “after” variant. Subsequently, relative abundances (ind./10 ha) were compared between before/after/not treated variants using the Kruskal–Wallis test (as assumptions of ANOVA were not met). These variants are also used in the following analysis, where relative abundances are compared between the described variants, divided into male/female, and analyzed separately. In this analysis, the Kruskal–Wallis test was also employed for the same reason as before.

For fawns, a separate analysis of repellent effects on the number of detected individuals (ind./10 ha) was performed. Linear regression was applied to analyze the trend in the numbers of detected individuals per 10 ha, depending on the time after application (in days). Independent models (linear regression fits) were created for plots after repellent applications and untreated ones. Only records after the last repellent application for a particular year (22 May 2023, and 18 May 2024) were included in the analysis to ensure comparable numbers of newborn fawns, assuming that the peak of roe deer fawning occurs after 20 May. For untreated plots, the time difference from the last repellent application was used for analysis. Records from one plot with an area of 1.07 ha with observed fawns were excluded because its abundance conversion to 10 ha was causing outlying values. The results are presented in the form of a scatter plot with fitted regression lines.

The relationship between adult abundance on research plots with repellent application and time since repellent application (in days) was assessed by linear regression. The “zero point” (i.e., the day of repellent application) was computed as the mean of all records before the day of repellent application. This value was set as a reference (100%) for each plot separately, and all other assessments were related to this value. Data were analyzed only for the first 10 days after repellent application, when repellents are considered the most effective (see above). The result of linear regression analysis is depicted in a scatter plot with a trend line.

All statistical procedures were performed in R software (version 4.3.2) [30], and plots were created using its package “ggplot2” [31]. For all statistical computations, an alpha level of 0.05 was set.

## 3. Results

**Figure 2:**
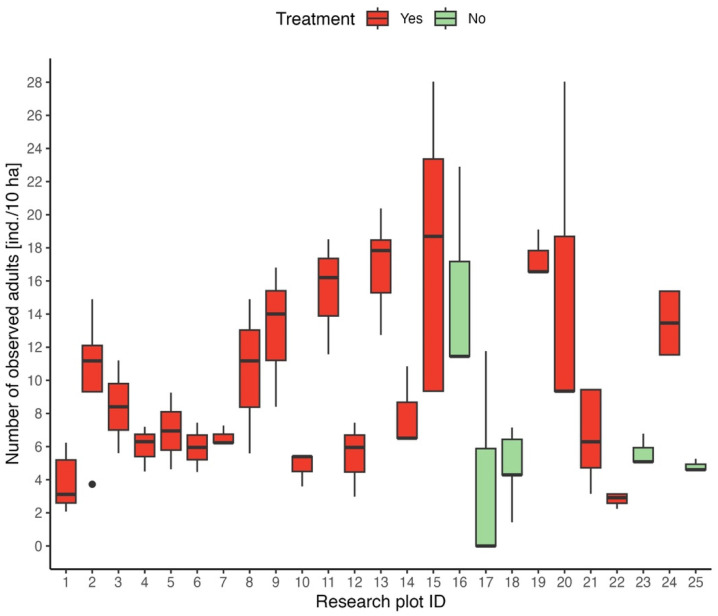
Boxplot of roe deer abundance for each research plot.

To gain insight into adult roe deer abundance on study plots, a boxplot of roe deer abundance was created for each study location, focusing on the time period prior to the application of the repellent. This serves as an initial visualization to assess the abundance across the dataset. The locations with/without repellent treatment are separated by different colors. Median abundance ranged from 0 ind./10 ha (mean = 0.57 ind./10 ha) on plot ID 17, whose area was 1.7 ha, to 18.7 ind./10 ha on plot ID 15, which area was 1.07 ha (Figure 2).

**Figure 3:**
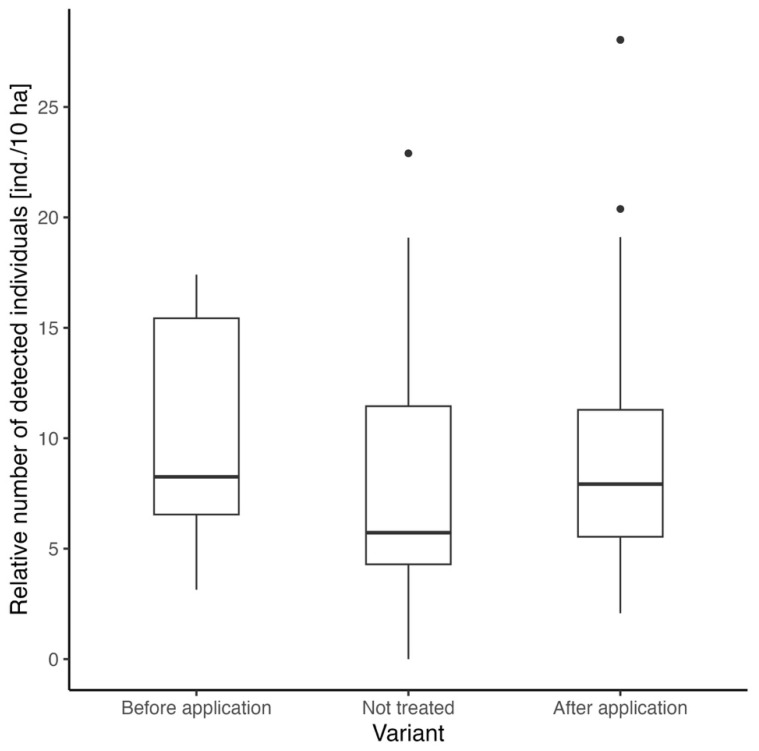
Comparison of the relative number of roe deer adults detected before repellent application, after repellent application, and on locations without any repellent treatment.

The assessment of the repellent effect on roe deer presence involved comparing the number of detected animals before and after the application of the repellent, alongside locations without any repellent treatment for benchmarking. All records before repellent application are considered as “before” and vice versa. The Kruskal–Wallis test shows marginal insignificance (chi-squared = 5.67, df = 2, *p* = 0.06). The highest median was observed for the “after application” variant (8.25 ind./10 ha), followed by “not treated” (7.92 ind./10 ha) and “before application” (5.72 ind./10 ha) variants (Figure 3).

**Figure 4:**
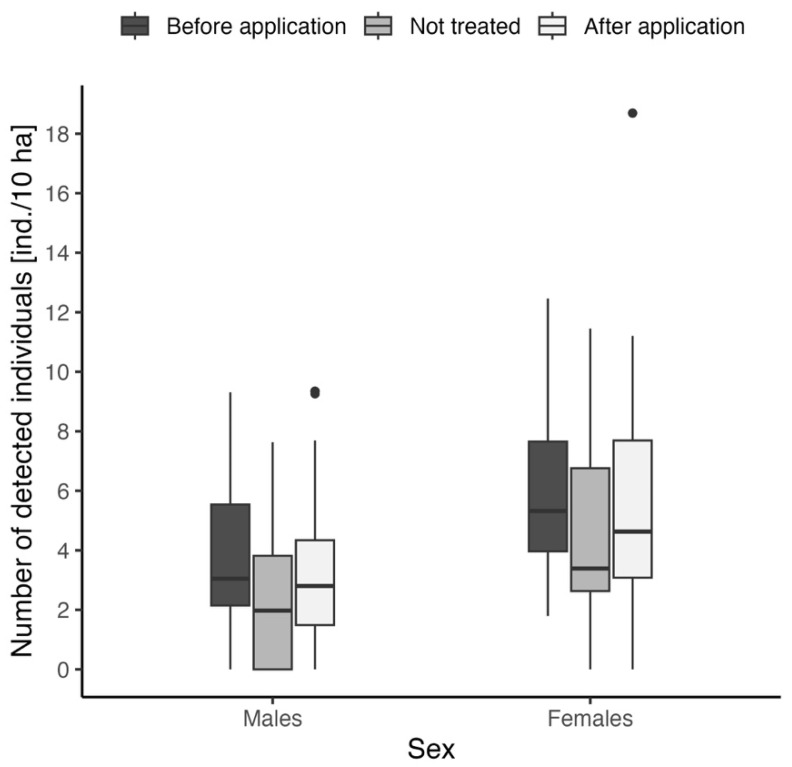
Comparison of the relative number of adults detected before/after repellent application and on locations without any repellent treatment, separated for adult males and females.

The comparison of roe deer presence before and after repellent application was also performed separately for adult males and females. In both cases, we used the Kruskal–Wallis test for comparison of numbers of detected individuals between before, after, and not treated variants (Figure 4). Individual records were considered “before” and “after” identically as in the previous case. In both cases, insignificant results were obtained: chi-squared = 3.96, df = 2, *p* = 0.14 for males and chi-squared = 3.68, df = 2, *p* = 0.16 for females. Males showed lower values of detected numbers in all cases (mean ± SD for “before application”: males—3.8 ± 2.7 ind./10 ha, females—6.0 ± 3.1 ind./10 ha; “after application”: males—3.7 ± 2.4 ind./10 ha, females—6.4 ± 4.1 ind./10 ha; “not treated”: males—2.5 ± 2.5 ind./10 ha, females—4.5 ± 3.7 ind./10 ha, see Figure 4).

**Figure 5:**
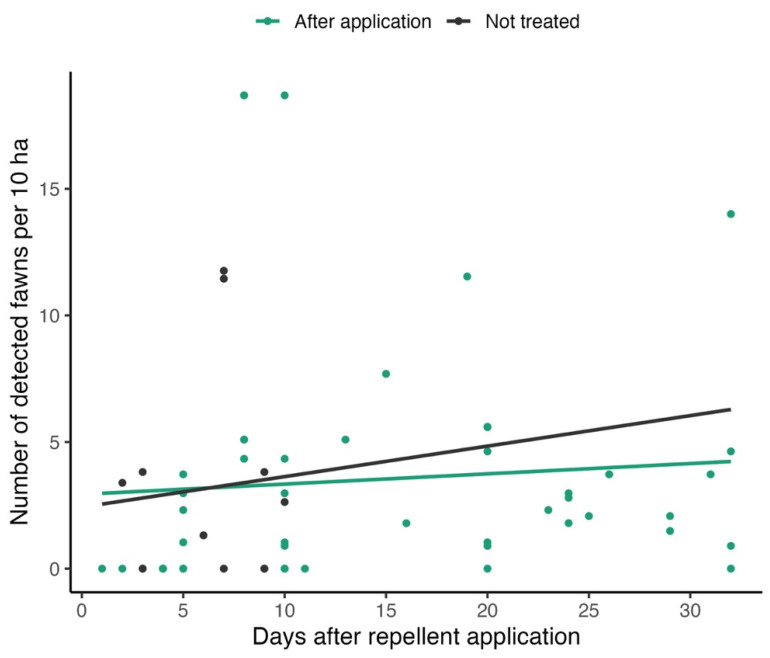
The relationship between relative numbers of detected fawns and time after repellent application (in days).

The dependence of relative numbers of detected fawns (ind./10 ha) on the time after repellent application was analyzed by linear regression (separately for plots after repellent application and untreated plots) (Figure 5). Only records after the last repellent application for a particular year were included in the analysis (see Section 2).

Both models showed extremely loose fits (R^2^ < 0.01 in both cases) with insignificant slope coefficients (*p* = 0.8 for untreated plots, *p* = 0.58 for plots after repellent application) showing high variability in relative abundance and an uncertain effect of repellents (the numbers of fawns did not change significantly after repellent application).

**Figure 6:**
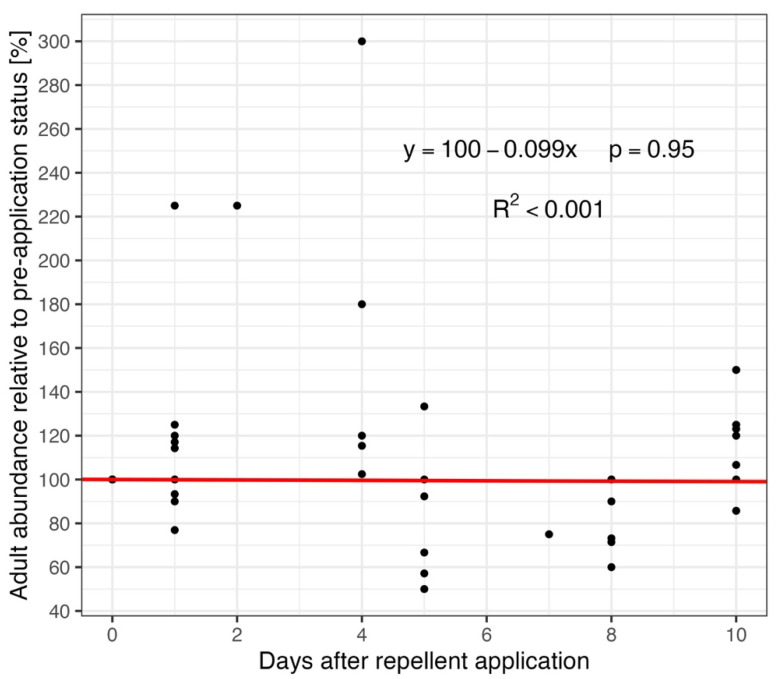
The relationship between adult abundance on research plots with repellent application and the time (days) after repellent application. The mean abundance recorded before the repellent was applied (day 0) was used as the reference value (set to 100%), and all subsequent values are expressed relative to this baseline. The red line depicts the result of linear regression (with fixed intercept of 100, as described in Section 2). Only ten days after the repellent application are selected for analysis, when the repellents are considered most effective (see Section 2).

Finally, the analysis of the development of adult abundance after repellent application was performed. All records before application were summarized, the mean abundance was computed, and this number was considered 100% (day 0—“repellent application day”). All other records were referenced to this value. Only data from the first 10 days following repellent application were included in the analysis, as repellents are considered effective within this period (see Section 2). No trend was observed using linear regression (y = 100 − 0.099x, *p* = 0.95, intercept of 100 was set as fixed, see Section 2)—repellent application did not have any consequence on roe deer abundance in the repellent effectivity time window, and R^2^ shows a very low value, suggesting an exceedingly loose fit (R^2^ < 0.001) (Figure 6).

## 4. Discussion

First, when evaluating the effectiveness of odor repellents for deflecting roe deer from risk locations, it is necessary to determine where the high population density is found. The proven numbers of adult individuals before the application of repellents ranged from 0 individuals/10 ha (median 0.57 individuals/10 ha) to 18.7 individuals/10 ha. According to data reported from northern Poland [32], a population density of 26 roe deer per 100 ha of forest area is considered high. Population densities in Italy ranged from 18.74 ind./10 ha to 29.0 ind./100 according to the counting method, as reported by Marcon et al. [33]. The incomparably higher roe deer density in our experiment is related to the small areas monitored. Here, roe deer are concentrated in meadows, which are one of the most preferred habitats in the time before giving birth to fawns due to high and dense vegetation [34,35,36,37].

After the application, the effect of the repellent was surprisingly the opposite of what was expected. The total number of adult game (regardless of sex) was the lowest before the application of the repellent, while subsequently, their numbers increased in the areas after the application, as well as in the locations without application. The differences between the values were non-significant. The slight increase in the number in the areas after the application and without application only indicates an increased number of roe deer in meadows before birthing. This is also confirmed by the analysis that compared the number of roe deer in the evaluated grasslands by sex. The number of females in the meadows before the application of the repellent (6.0 ± 3.1 ind./10 ha) significantly exceeds the number of males (3.8 ± 2.7 ind./10 ha), which may again indicate the habitat preferences of females towards meadows and the subsequent laying of young in such areas. The significant disparity between males and females may also be due to the local hunting practice, which, in Central Europe, is aimed at hunting trophy game, and that can skew the ungulate population in favor of females in the long term [17]. The results also show non-significant differences between the variants before and after application of the repellent compared to untreated areas, where the sex ratio did not fundamentally change after application.

The main objective of the repellent testing was, however, primarily to determine the effectiveness of the expected expulsion of roe deer fawns from risk areas. The results indicated no substantial differences in fawn detection between treated and untreated areas. The number of fawns in the period after the last application of the repellent, which occurred on 22 May 2023, and similarly, on 18 May 2024, constantly increased regardless of the repellent treatment or non-treatment option. The increase in the number of fawns found is related to the date of their birth. The end of repellent application period was, in general, at the beginning of the time when most of the fawns are born, according to Jarnemo et al. [38], who reports that about two-thirds of fawns are born between 25 May and 7 June, or with Christen et al. [35], who report the mean date of birth on 22 May. Therefore, it is evident that the number of fawns detected in the meadows will be higher at the beginning of June compared to May.

The latest analysis compared the relative number of adult deer in the areas after repellent treatment. The assumption was a reduction in the number of adult deer, which is expected to persist for several weeks according to the manufacturer, but it should probably be clear immediately after application, when the odor repellent is released from the carrier most strongly. However, the number of adult deer in the areas did not change in any way, which again indicates the ineffectiveness of odor repellents for the purpose of expelling deer from meadows before mowing. These findings correspond with previously published papers. For example, Elmeros et al. [23] tested odor repellents on the avoidance area of roe and red deer (*Cervus elaphus*). Their study confirmed no significant reduction in the visitation of both cervidae species after applying two types of odor repellents to forest stands. Other studies have focused on odor repellent use in deterring wild boar (*Sus scrofa*). The experiment by Schlageter and Haag-Wackernagel [39] aimed to evaluate the wild boar preference and recorded a minimal and non-significant deterrent effect of attendance at feeding sites when one of the pair of sites was treated with an odor repellent. Both feeding sites were visited at almost the same frequency; thus, the authors concluded that the repellent is an ineffective measure for crop protection against wild boar damage. The ineffectiveness of odor repellents was also confirmed by a recently published study evaluating the effect of an odor barrier on wild boar movement. Wild boar individuals were tracked by telemetry collars for 22 days, after which a line of odor repellents was installed along their commonly used corridor between feeding and resting sites. GPS telemetry revealed that the movements of the monitored animals did not change after the repellents were applied and remained the same as before application [40].

Based on the provided results and previously published studies, it is apparent that in the case of roe deer fawns’ protection against agricultural machinery, it is necessary to apply another type of measure. For instance, the utilization of UAVs equipped with thermal cameras seems to be a suitable solution with up to 100% efficiency [15]. However, these methods are predominantly aimed only at roe deer fawns’ protection with no interest in small animals, ground-nesting birds, or even invertebrates. Therefore, we would like to highlight the necessity for complex solutions, which could be provided by an increase in extensively used agricultural areas, like wildflower strips or set-aside land. In general, food supply and suitable cover options [25,41] contribute a key factor influencing habitat selection [42,43,44]. In the context of roe deer, suitable areas can allow for relatively small home ranges [36,45] if there is a high-quality food supply. Sufficient cover and area often become long-term staging. Ultimately, habitat availability critically determines where a roe deer female places newborn fawn. This was confirmed, e.g., in Switzerland, where roe deer fawns were found in grassland (71%), but also in forest habitats (23%) and in fields (6%), with a preference for forest edges and localities with a medium vegetation height of 20–50 cm [35]. However, in the poor habitats of central Norway, a substantial share of roe deer fawns preferred moorland over meadow, which again points to habitat availability [46]. In this habitat context, it is necessary to mention that the average size of meadows with the odor repellent application was 5.52 ha ± 2.47 SD. The year-round roe deer home range size extends over an area of several tens of hectares [47,48,49]. During the spring, it was, for example, 28.8 ha for roe deer females in the mixed habitats in France [50]. Therefore, within the home range is wide range of habitats where roe deer fawns could be born or could be used as resting places in the neighborhood of meadows affected by odor repellent application. Given sufficient availability of suitable habitats in the landscape, a substantial reduction in losses of roe deer fawns caused by agricultural machinery can therefore be expected. The roe deer habitat preference is known for agri-environmental measures consisting of wildflower strips and extensive orchards [51] which could mitigate roe deer fawn mortality due to no harvest within the spring season.

However, the preference for biodiversity-rich measures such as wildflower strips and other purpose-designed habitats should be subjected to further rigorous verification in the context of wider ecosystems. The wider context of the locality could also affect the odor repellent effectiveness. Climatic conditions and local wind may also affect repellent effectiveness. Here, the main aim was to point out the effectiveness of odor repellent, in general, as the previously published research indicated low effect on wild ungulates [25]. Another limitation of our study could be the lower number of research plots included in the study. Nevertheless, we have observed a clear trend of no repellent application and therefore, we have decided to evaluate the data to disseminate the findings so that, in the future, roe deer fawns will not continue to be killed by agricultural machinery due to an ineffective protection method and to encourage the use of alternative measures such as thermal-imaging drones or habitat management.

## 5. Conclusions

The evaluation of odor repellent applications used to drive roe deer out of fields before meadow harvest indicated that the measure is ineffective for adult animals of both sexes and also for roe deer fawns. In both cases, comparable numbers of fawns and adults were still present on treated areas after application compared to meadows without odor deterrents. The experiment confirmed the generally low efficacy of odor repellents for displacing roe deer individuals (juveniles and adults) from high-risk fields, in the short-term (days), as well as over longer periods (weeks). The study, therefore, highlights the need to use alternative methods, such as drones equipped with thermal imaging, whose high effectiveness has already been demonstrated. It is, however, crucial to implement comprehensive, ecosystem-level solutions that protect not only roe deer fawns but also other fauna—vertebrates and invertebrates alike. There is a lack of research on roe deer habitat preferences and the locations where fawns are laid down during the haymaking period in altered landscapes with a high proportion of non-productive areas. This scientific gap is indicating the high need for further research aimed at safeguarding the entire agroecosystem rather than selected species alone.

## Data Availability

The Dataset is available on request from the authors.
